# Auricular discoid lupus erythematosus

**DOI:** 10.1016/j.jdcr.2026.03.039

**Published:** 2026-03-27

**Authors:** Zachary J. Jaeger, Brian R. Hinds, Marleigh J. Stern

**Affiliations:** Department of Dermatology, University of California San Diego, San Diego, California

**Keywords:** alopecia, anti-beta-2-microglobulin antibodies, antinuclear antibodies, anti-Sjögren syndrome-A antibodies, arthralgia, auricular, biopsy, concha, cymba, dermatitis, dermatoscope, dermoscopy, discoid lupus erythematosus, ear, follicular plugging, histopathology, hydroxychloroquine, hyperpigmented, hypocomplementemia, interface, perifollicular fibrosis, photosensitivity, pigment incontinence, scarring, smoking, systemic lupus erythematosus, telangiectasias, tobacco, topical steroids

## Clinical presentation

**Fig 1:**
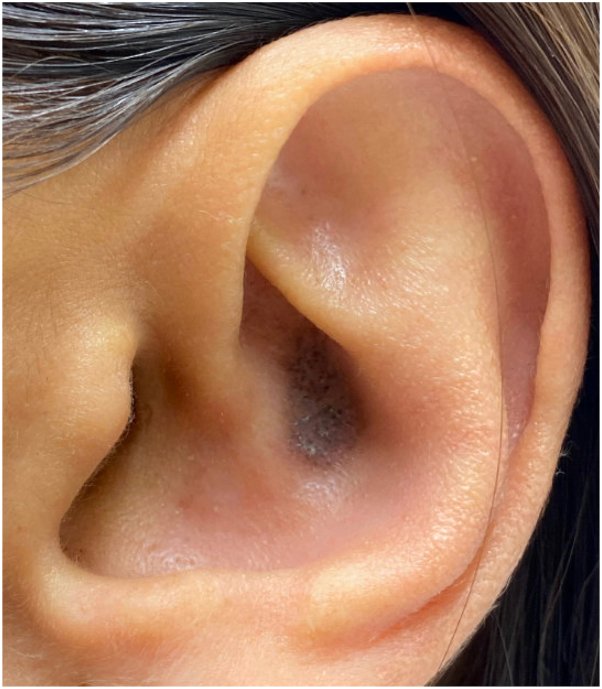
Auricular discoid lupus erythematosus: a hyperpigmented, slightly scaly plaque in the cymba concha.

A 43-year-old woman presented with a solitary, well-demarcated, hyperpigmented plaque on the cymba concha (Fig 1). The patient reported photosensitivity and diffuse arthralgias and history of tobacco use. A thin shave biopsy was performed.

## Dermoscopic appearance

**Fig 2:**
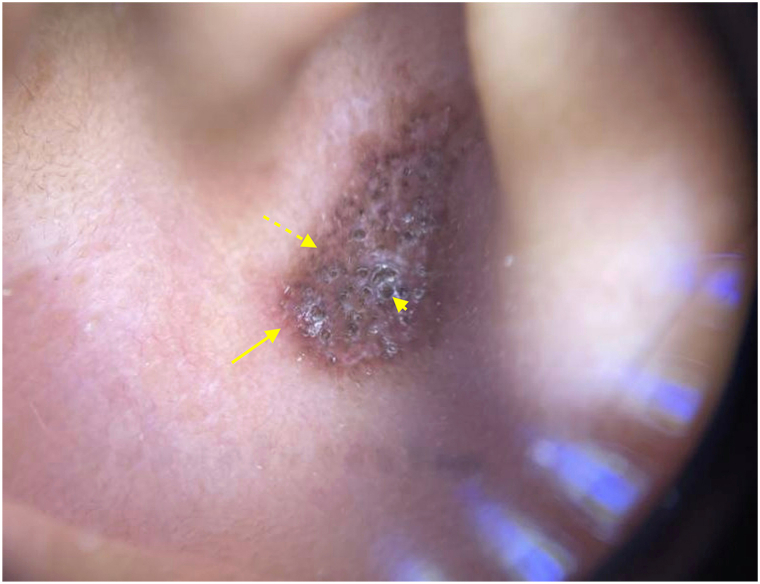
Auricular discoid lupus erythematosus on dermoscopy: an erythematous, hyperpigmented (*dashed arrow*) plaque with diffuse, dilated, plugged follicles (*short arrow*), peripheral telangiectasias (*long arrow*), focal fine scale, whitish structureless zones, and hypopigmented dots.

Dermoscopy revealed extensive, patulous follicular plugging (short arrow), erythema and telangiectasias most notable at the periphery (long arrow), with diffuse hyperpigmentation (dashed arrow) and areas of fine scale, whitish structureless zones, and hypopigmented dots (Fig 2).

## Histologic diagnosis

Histopathology demonstrated a brisk interface dermatitis encircling the hair follicles, along with pronounced basement membrane thickening and pigment incontinence, consistent with the diagnosis of discoid lupus erythematosus (DLE).

Key message

**Fig 3:**
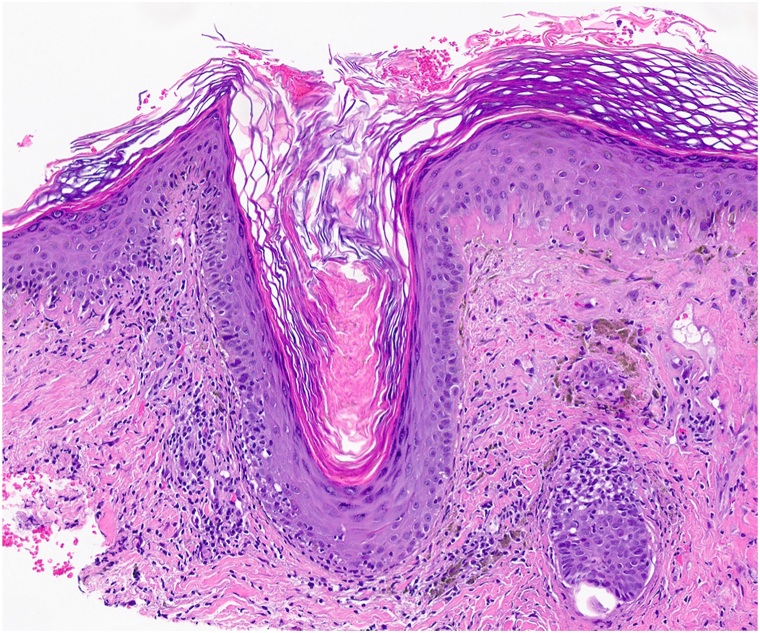
Auricular discoid lupus erythematosus on histopathology (hematoxylin & eosin stain, 100× magnification): notable hyperkeratotic follicular plugging, perifollicular interface dermatitis, basement membrane thickening, melanin incontinence, and dermal fibrosis.

Characteristic dermoscopic features (and histopathologic correlates) in DLE include follicular keratotic plugs and dilated follicular openings (corresponding to follicular hyperkeratosis with microscopy), perifollicular whitish halo (perifollicular fibrosis), dilated superficial vessels (telangiectasias), white scale (hyperkeratosis), pigmentation (pigmentary incontinence), structureless white areas (dermal fibrosis), and follicular red dots (perifollicular erythrocyte extravasation) (Fig 3).^1^^,^^2^ Prominent pigmentary incontinence may mask other findings in skin of color, while hyperkeratotic follicular plugging with background erythema and telangiectasia is more apparent in lighter skin. Location in an auricular concavity and direct dermoscopic examination raised suspicion for DLE.

Subsequent rheumatologic work-up was pertinent for positive antinuclear antibodies, anti-Sjögren syndrome-A antibodies, low-titer anti-beta-2-microglobulin antibodies, and hypocomplementemia in the absence of synovitis or arthritis, not meeting criteria for systemic lupus erythematosus. The patient initiated oral hydroxychloroquine 300 mg daily (∼5 mg/kg/day) and desonide 0.05% ointment to the ear lesion with improvement in appearance. Smoking cessation and diligent photoprotection were encouraged.

## Conflicts of interest

None disclosed.
